# Association between MMP-2 gene polymorphism and cataract susceptibility: A protocol for systematic review and meta-analysis

**DOI:** 10.1097/MD.0000000000025392

**Published:** 2021-04-09

**Authors:** Huaiyan Jiang, Yang Gao, Zhen Chen, Hongxia Xu

**Affiliations:** Department of Ophthalmology, The Affiliated Kunhua Hospital of Kunming University of Science and Technology, The First People's Hospital of Yunnan Province, Kunming 650032, Yunnan Province, China.

**Keywords:** cataract, matrix metalloproteinase-2, meta-analysis, polymorphism, protocol

## Abstract

**Background:**

Matrix metalloproteinase-2 (MMP-2) polymorphisms have been considered as risk factors of cataracts, but the results still remain controversial. In this study, we have performed a systematic meta-analysis to evaluate the association between MMP-2 polymorphisms and cataract risks.

**Methods:**

Published literature was retrieved from Wanfang, Chinese Biomedical Literature Database, Chinese National Knowledge Infrastructure, Chongqing VIP Chinese Science and Technology Periodical Database, PubMed, Embase, and Web of Science databases. The case–control studies that explored the association between MMP-2 polymorphisms and cataract risks were included. Pooled odds ratio (OR) and 95% confidence interval (CI) were calculated using random- or fixed-effects model.

**Results:**

This study could provide high-quality and evidence-based medical evidence for the correlation between MMP-2 polymorphisms and cataract risks

**Conclusion:**

The study could provide updated evidence for the evaluation of the relationship between MMP-2 polymorphism and cataract risk.

**Ethics and dissemination:**

The private information from individuals will not be published. This systematic review also will not involve endangering participant rights. Ethical approval is not available. The results may be published in a peer-reviewed journal or disseminated in relevant conferences.

**OSF Registration Number:**

DOI 10.17605/OSF.IO/KU9NE.

## 1 Introduction

Cataract is caused by lens opacity due to the destruction of crystalline microstructure, which adversely affects the transmission of light on the retina.^[1]^ Currently, cataract is commonly lead to blindness in the world, accounting for 46% of the world's blindness.^[2]^ Epidemiological studies have displayed that ultraviolet radiation, ionizing radiation, smoking, and the use of steroids are risk factors of cataracts.^[3]^ In recent years, it has been found that genetic factors play an important role in the pathogenesis of cataract.^[4]^ In addition, according to reports, gene polymorphism is associated with the risk of cataract.^[5,6]^

The etiology of cataract is complicated. Meanwhile, it is generally believed that the study of gene polymorphism may reveal the nature of cataract.^[7–9]^ As a new molecular marker, single nucleotide polymorphism plays significant in the pathogenesis of cataract.^[10]^ The in-depth study on the relationship between gene single nucleotide polymorphism and cataract is helpful to clarify the occurrence of cataract at the molecular level.

Matrix metalloproteinases (MMPs) refer to a class of proteolytic enzymes that can hydrolyze extracellular matrix.^[11,12]^ Based on some researches, the invasion and metastasis of cells are related to the degradation of extracellular matrix.^[13,14]^ As an important member of the MMPs family, matrix metalloproteinase-2 (MMP-2) is a proteolytic enzyme based on type IV collagen and laminin, which can specifically degrade the matrix components of normal lens capsule.^[15,16]^ The increase of MMP-2 concentration can enhance the degradation of extracellular matrix, promote cell proliferation and migration, and transform mesothelial cells into myofibroblasts.^[17,18]^ Finally, it could result in matrix contracture, cell aggregation, collagen deposition, posterior capsule wrinkling, and opacity.^[19,20]^ Therefore, MMP-2 protein that is formed after the transcriptional translation of MMP-2 gene may be related to the occurrence and development of cataract.^[21,22]^

At present, the research on the relationship between MMP-2 and cataract has become a hot spot, and especially at the gene level, the conclusions about MMP-2 gene polymorphism and cataract susceptibility are not consistent.^[23]^ The purpose of this study is to explore the relationship between MMP-2 gene polymorphism and cataract susceptibility through meta-analysis, thus providing a new direction for early prevention and treatment of cataract.

## 2 Methods

### 2.1 Study registration

The protocol of the systematic review was registered on Open Science Framework, and the registration number is DOI 10.17605/OSF.IO/KU9NE. This meta-analysis protocol was on the basis of the Preferred Reporting Items for Systematic Reviews and meta-analysis Protocols (PRISMA-P) Statement Guidelines.^[24]^

### 2.2 Data sources and search strategy

We carried out a literature search in Wanfang, Chinese Biomedical Literature Database, Chinese National Knowledge Infrastructure, Chongqing VIP Chinese Science and Technology Periodical Database, PubMed, Embase, and Web of Science databases up to February 2021, with the following MeSH terms and keywords “cataract,” “matrix metalloproteinase-2,” and “polymorphism.” The search strategy for PubMed is listed in Table 1.

**Table 1:** Search strategy in PubMed database.

Number	Search terms
#1	Matrix metalloproteinase 2[MeSH]
#2	Gelatinase A[Title/Abstract]
#3	72-kDa Gelatinase[Title/Abstract]
#4	72-kDa Type IV Collagenase[Title/Abstract]
#5	MMP-2 Metalloproteinase[Title/Abstract]
#6	MMP2 Metalloproteinase[Title/Abstract]
#7	Matrix Metalloproteinase-2[Title/Abstract]
#8	72 kDa Gelatinase[Title/Abstract]
#9	72 kDa Type IV Collagenase[Title/Abstract]
#10	Gelatinase, 72-kDa[Title/Abstract]
#11	MMP 2 Metalloproteinase[Title/Abstract]
#12	Metalloproteinase 2, Matrix[Title/Abstract]
#13	Metalloproteinase, MMP-2[Title/Abstract]
#14	Metalloproteinase, MMP2[Title/Abstract]
#15	MMP-2[Title/Abstract]
#16	or/1–15
#17	Cataract[MeSH]
#18	Cataract, Membranous[Title/Abstract]
#19	Lens Opacities[Title/Abstract]
#20	Pseudoaphakia[Title/Abstract]
#21	Cataracts[Title/Abstract]
#22	Cataracts, Membranous[Title/Abstract]
#23	Lens Opacity[Title/Abstract]
#24	Membranous Cataract[Title/Abstract]
#25	Membranous Cataracts[Title/Abstract]
#26	Opacities, Lens[Title/Abstract]
#27	Opacity, Lens[Title/Abstract]
#28	Pseudoaphakias[Title/Abstract]
#29	or/17–28
#30	polymorph∗[Title/Abstract]
#31	susceptibility[Title/Abstract]
#32	or/30–31
#33	#16 and #29 and #32

### 2.3 Inclusion criteria for study selection

The inclusion criteria for eligible studies in this meta-analysis are as follows:

(a) a study evaluating the association between MMP-2 polymorphism and cataract,
(b) a case–control study,
(c) a study with available genotype frequency, and
(d) a study with sufficient data for the estimation of odds ratio (OR) and 95% confidence interval (CI).

Exclusion criteria are as follows: animal studies; reviews; conference abstracts; and letters to the editors. For studies performed in the same patients, only the study with the largest sample size was included. The corresponding authors of all eligible studies were not contacted for further information.

### 2.4 Data collection and analysis

#### 2.4.1 Selection of studies

**Figure 1:**
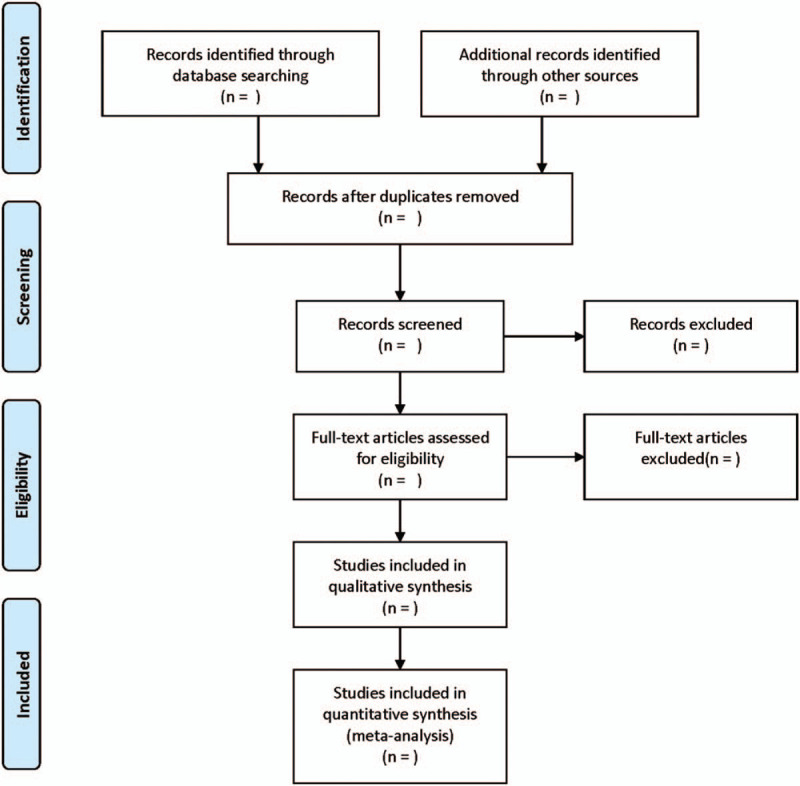
Flow diagram of study selection process.

According to the PRISMA flowchart (Fig. 1), all reviewers received evidence-based training and adhered to the summarized process. According to the inclusion and exclusion criteria, 2 researchers will independently introduce the literature into EndNote X7, set up a group, check the repetition, screen the literature that obviously does not meet the requirements through reading topics and abstracts, and screen the literature that meets the requirements again after reading the full text. It is difficult to determine whether it is included or not for the differences, and then the third researcher would discuss and decide whether it will be included or not.

#### 2.4.2 Data extraction and management

Two investigators independently assessed the articles for inclusion, and reached a consensus on extracted data. For each study, the following information was extracted: the first author name and publication year of the article; ethnicity (country) of study subjects; gene polymorphisms and genotype frequencies; sample size (numbers of cases and controls); sources of controls; subtypes of cataract classified. Hardy–Weinberg equilibrium (HWE) in controls was calculated as previously described.

### 2.5 Assessment of quality in included studies

The quality of all the included studies will be evaluated by 2 reviewers independently based on the Newcastle–Ottawa scale (NOS) that is applied to evaluate the quality of observational studies.^[25]^ Disagreement will be reported and resolved by a third reviewer. The NOS values arrange from 0 to 9. Studies with the score of 6 are considered to be of high quality.

### 2.6 Dealing with missing data

The reason for the loss of data in the period of data screening and extraction is identified here. If the data of potential studies are insufficient, missing, or vague, we would attempt to contact the authors. Only if the data are not available through the method described above, these studies would be excluded.

### 2.7 Statistical analysis

The HWE for control subjects of each studies was evaluated by carrying out a chi-square test, and *P* < .05 was seen as significant disequilibrium. The association between MMP-2 polymorphism and cataract was estimated by calculating pooled OR subtype. The pooled ORs were executed for homozygote comparison, dominant and recessive models, allele comparison and heterozygote comparison. The heterogeneity was calculated by performing the chi-square-based *I*^2^ test and the *Q* test. The fixed-effect model (the Mantel–Haenszel method) was chosen when the *I*^2^ value is less than 50%. While *I*^2^ was more than 50%, a random-effects model (DerSimonian and Laird method) was adopted. All of the statistical analyses were conducted by the STATA 16.0 (StataCorp, College Station, TX), and the *P* values were two-sided.

### 2.8 Subgroup analysis

According to different ethnicity, genotyping method, and so on, we conducted subgroup analyses on the relationship between MMP-2 gene polymorphisms and the risk factors of cataract.

### 2.9 Sensitivity analysis

When 1 single study was excluded each time, the one-way sensitivity analysis was performed and the new-pooled results that reflect the influence of the study deleted to the overall OR.

### 2.10 Assessment of publication biases

Publication bias was assessed by Begg's rank correlation and Egger's linear regression, and was seen to be statistically significant when *P* < .05.^[26,27]^

### 2.11 Ethics and dissemination

The content of this article does not involve moral approval or ethical review and would be presented in print or at relevant conferences.

## 3 Discussion

Some studies have illustrated that abnormal gene expression is closely related to the occurrence of cataract, which can lead to changes in the transcription and translation of various proteins in the lens, thus damaging the structure and function of the lens, and eventually leading to cataract.^[28–30]^ Therefore, the search for lens-specific genes is of great significance to explore the pathogenesis of cataract.

As a calcium and zinc dependent proteolytic enzyme, MMPs play an important role in embryonic development, wound repair, cell migration, and so on.^[31]^ MMP-2 affects the migration, shuttle, proliferation and growth of lens posterior capsule cells, and is closely related to the occurrence and development of cataract.^[32,33]^ In this paper, meta-analysis was carried out to systematically analyze the relationship between multiple mutation sites of MMP-2 and cataract susceptibility, which will provide evidence-based medicine for early prevention and treatment of cataract.

This meta-analysis has some limitations. First of all, cataract is a multifactorial disease, and other confounding factors, including ultraviolet radiation and smoking, may also affect the associated analyses. However, the included study did not provide a detailed record of these confounding factors. As a result, the link we found in the review may be strengthened or weakened by these confounding factors. We hope that future researches will pay more attention to these effects if possible. Second, the occurrence of cataract is not a single molecular event. Only when multiple genes work together, the risk of cataract can be increased. Genetic factor is one of the risk factors of cataract. Strengthening the study of the interaction between gene and environmental factors will be more helpful to deepen the understanding of cataract and provide a new therapeutic target for disease prevention and treatment at the genome level to maximize the quality of life of patients.

## Author contributions

**Conceptualization:** Hongxia Xu, Huaiyan Jiang.

**Data curation:** Huaiyan Jiang, Yang Gao.

**Funding acquisition:** Hongxia Xu.

**Investigation:** Hongxia Xu.

**Methodology:** Yang Gao.

**Project administration:** Hongxia Xu.

**Resources:** Yang Gao.

**Software:** Yang Gao, Zhen Chen.

**Supervision:** Yang Gao, Zhen Chen.

**Validation:** Zhen Chen.

**Visualization:** Zhen Chen.

**Writing – original draft:** Hongxia Xu, Huaiyan Jiang, Zhen Chen.

**Writing – review & editing:** Hongxia Xu, Huaiyan Jiang.
